# Investigation of Tick-Borne Pathogens in *Ixodes* Ticks from Bosnia and Herzegovina

**DOI:** 10.3390/ani14152190

**Published:** 2024-07-27

**Authors:** Jasmin Omeragić, Naida Kapo, Šejla Goletić, Adis Softić, Ilma Terzić, Emina Šabić, Vedad Škapur, Darinka Klarić Soldo, Teufik Goletić

**Affiliations:** 1 Veterinary Faculty, University of Sarajevo, Zmaja od Bosne 90, 71000 Sarajevo, Bosnia and Herzegovina; jasmin.omeragic@vfs.unsa.ba (J.O.); sejla.goletic@vfs.unsa.ba (Š.G.); adis.softic@vfs.unsa.ba (A.S.); ilma.terzic@vfs.unsa.ba (I.T.); eminasabic123@hotmail.com (E.Š.); darinka.klaric.soldo@vfs.unsa.ba (D.K.S.); teufik.goletic@vfs.unsa.ba (T.G.); 2 Faculty of Agriculture and Food Science, University of Sarajevo, Zmaja od Bosne 8, 71000 Sarajevo, Bosnia and Herzegovina; v.skapur@ppf.unsa.ba

**Keywords:** *Ixodes ricinus*, *Ixodes canisuga*, *Ixodes hexagonus*, *Babesia* spp., *Anaplasma phagocytophilum*, *Rickettsia* spp., *Borrelia burgdorferi* s.l.

## Abstract

**Simple Summary:**

Ticks are ectoparasites with medical significance. They inhabit diverse environments and maintain close interactions with numerous vertebrate hosts. *Ixodes* ticks can transmit various pathogens to animals and humans. The aim here was to examine *Ixodes* ticks from Bosnia and Herzegovina to check for specific pathogens. This study found *Rickettsia* spp., *Babesia* spp., *Anaplasma phagocytophilum*, and *Borrelia burgdorferi* sensu lato in ticks from domestic animals. These findings highlight the need for the ongoing monitoring of ticks and tick-borne pathogens to protect animal and public health. Additionally, this study provides valuable insights into the occurrence and spread of these pathogens, emphasizing the importance of broader surveillance and control measures. Effective prevention, surveillance, and control of tick-borne diseases require urgent regional and international collaboration.

**Abstract:**

Limited information is available regarding the presence of tick-borne pathogens and their distribution within *Ixodes* species in Bosnia and Herzegovina. This study aimed to identify *Rickettsia* spp., *Babesia* spp., *Anaplasma phagocytophilum*, and *Borrelia burgdorferi* sensu lato (s.l.) in *Ixodes* ticks collected from domestic and wild animals and vegetation in different regions across Bosnia and Herzegovina. A total of 7438 adult ticks, including 4526 *Ixodes ricinus*, *Ixodes canisuga*, and *Ixodes hexagonus*, were collected. Real-time PCR screening of 450 pooled *I. ricinus* samples revealed a 22.1% infection rate with at least one pathogen. *Rickettsia* spp. (6.3%) were found in ticks from dogs, cats, and goats, *Babesia* spp. (3.1%) in ticks from dogs and cattle, *A. phagocytophilum* (8.8%) in ticks from dogs, goats, and cattle, and *B. burgdorferi* s.l. (3.4%) in ticks from dogs and cats. Mixed infections with *B. burgdorferi* s.l. and *A. phagocytophilum*, as well as *B. burgdorferi* s.l. and *Rickettsia* spp., were found in two pools of *I. ricinus* from dogs and cats, respectively. Additionally, co-infection with *Rickettsia* spp. and *A. phagocytophilum* was confirmed in three tick pools from dogs and goats. Each tick from these pooled samples was individually retested to confirm the presence of pathogens. In the examined pooled samples of *I. canisuga* (1) and *I. hexagonus* (6), none of the tested pathogens were detected. Our findings represent the first detection of *Rickettsia* spp., *Babesia* spp., *A. phagocytophilum*, and *B. burgdorferi* s.l. in *I. ricinus* collected from domestic animals and vegetation in Bosnia and Herzegovina. Considering the established infection rates, the detection of tick-borne pathogens in adult ticks collected from domestic animals and vegetation enriches the current knowledge of the presence of tick-borne pathogens at the local, regional, national, and broader levels.

## 1. Introduction

Alongside mosquitoes, ticks are considered the primary vectors of the most important infectious diseases in humans and animals worldwide [1]. In recent years, the spread of tick-borne pathogens (TBPs) has increased, and the diseases they transmit are appearing in new regions or re-emerging within endemic areas, posing growing concerns for veterinary and public health, as well as biodiversity conservation [2]. Evidence indicates that zoonotic tick-borne diseases (TBDs) are expanding their geographical range, with infection rates posing a potential future public health crisis [3]. Given these factors, the study of TBPs and their occurrence in different tick species is crucial for understanding TBDs in humans, livestock, pets, and wildlife [3]. 

In Europe, *Ixodes ricinus* is the most widely distributed species, with its range extending across the continent and infesting numerous animal species and humans [4]. It is considered a serious health concern due to its extensive host population and ability to transmit various pathogens, including the tick-borne encephalitis virus (TBEV), *Borrelia burgdorferi* sensu lato (s.l.), the causative agent of Lyme borreliosis, and other pathogens such as *Anaplasma phagocytophilum*, as well as the pathogenic species of *Rickettsia*, *Bartonella*, *Francisella*, and *Babesia* [5,6,7,8]. In most European countries, *Ixodes canisuga* is commonly found infesting medium-sized Mustelidae and Canidae, such as badgers (*Meles meles*), jackals (*Canis aureus*), red foxes (*Vulpes vulpes*), and European hedgehogs (*Erinaceus europaeus*) [9,10]. Like *I. canisuga*, *Ixodes hexagonus* is also distributed throughout Europe and is associated with hedgehogs, *Erinaceus europaeus*, and *Erinaceus roumanicus*, as well as a wide range of medium-sized burrow-inhabiting mammals such as red foxes, badgers, European pine martens (*Martes martes*), stoats (*Mustela erminea*), European polecats (*Mustela putorius*), and occasionally otters (*Lutra lutra*) [9,11]. However, both species have been recorded biting humans and their companion animals; for example, *I. canisuga* is often found feeding on dogs, while *I. hexagonus* commonly feeds on pet cats and dogs and less frequently on humans [12,13,14]. Furthermore, pathogens such as *Rickettsia*, *A. phagocytophilum*, *Babesia*, and *B. burgdorferi* s.l. have been confirmed in both *I. canisuga* and *I. hexagonus* [15,16,17,18,19,20].

In previous studies conducted in Bosnia and Herzegovina, the presence of four tick species from the genus *Ixodes* has been recorded, namely *I. ricinus*, *I. canisuga*, *I. hexagonus*, and *Ixodes vespertilionis* [21,22,23,24,25]. Previous research has confirmed the dominant presence of *I. ricinus* [22,24,25] collected from domestic and wild animals, with a prevalence of 70.5% and 63.8% in 2008 and 2022, respectively. *Ixodes canisuga* and *I. hexagonus* were found infesting animals (primarily dogs and red foxes), with prevalence rates of 0.3% and 0.1%, respectively [24,25,26]. In Bosnia and Herzegovina, there are data on pathogens confirmed in *I. ricinus* in several studies, where the presence of *B. burgdorferi* s.l. complex has been confirmed in ticks collected from humans [27]. Additionally, in *I. ricinus* collected from animals, the presence of *Babesia divergens*, *Rickettsia monacensis*, *Rickettsia Helvetica*, and TBEV has been established [28,29,30]. Furthermore, no other studies have been encountered that reveal the presence of TBPs in other *Ixodes* species. 

Data on TBPs in Bosnia and Herzegovina are limited. Therefore, this study aims to investigate the presence and distribution of *Ixodes* ticks and to identify pathogens including *Rickettsia* spp., *Babesia* spp., *A. phagocytophilum*, and *B. burgdorferi* s.l. in ticks collected from domestic and wild animals as well as from vegetation across diverse regions of Bosnia and Herzegovina.

## 2. Materials and Methods

### 2.1. Study Area and Tick Collection

**Figure 1:**
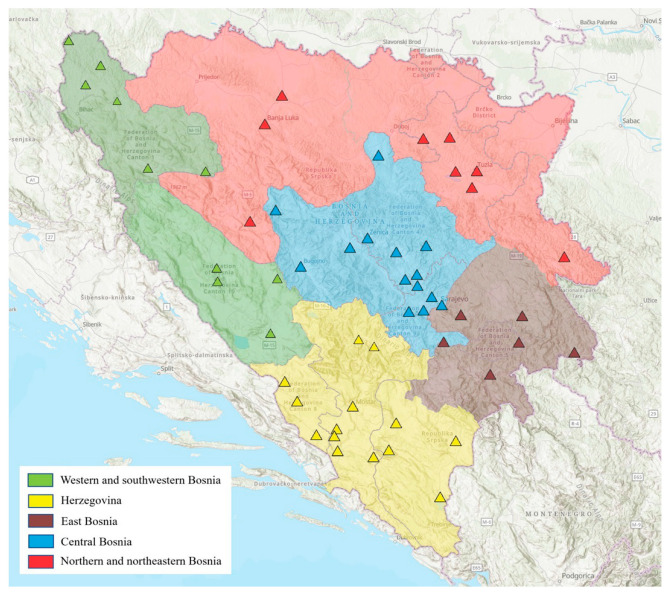
Geographical overview of the regions and sampling sites of Bosnia and Herzegovina included in the study. The study map was developed using ArcGIS^®^ Pro software (ESRI, Redlands, California, United States of America), version 3.2.2.

The collection of ticks was conducted across the entire territory of Bosnia and Herzegovina from 2017 to 2023. The methods of tick collection and sampling procedures were carefully tailored to match the geographical and bioclimatic characteristics of the study area. Sampling was based on prior knowledge of tick distribution and abundance in Bosnia and Herzegovina, as depicted in Figure 1.

The sampling of ticks involved the direct collection of adult ticks from domestic and wild animals by veterinarians and animal owners in defined areas. The adult ticks were carefully isolated from the host body using tweezers to avoid any external damage to the specimens. Collection of adult ticks from vegetation, leaf litter, or other substrates was conducted once between May and August by dragging a white flannel blanket [31] over the leaf litter and low vegetation near farms and enclosures where the animals from which ticks were previously sampled resided. Ticks were then collected from the fabric using forceps or a tick removal tool. All tick species encountered, whether questing or feeding, were collected without any specific selection using the previously described sampling methods (n = 7438).

### 2.2. Morphological Identification of Ticks and Tick Pooling Procedure

After collection, the adult ticks were stored refrigerated at +4 °C and transported to the Laboratories of the University of Sarajevo-Veterinary faculty (accredited by BAS EN ISO/IEC 17025:2018 [32]). The ticks were counted, pooled, and identified based on morphological characteristics using a Zeiss Stemi 508 stereomicroscope (magnification 0.63–5.0×), following established identification keys [33,34].

Once the ticks were morphologically identified (7438), only *Ixodes* species (4526) were selected for this study. Subsequently, adult ticks were grouped and classified according to various characteristics such as engorgement status, sex, sampling location, vegetation, and host from which they were collected. Of the collected adult *Ixodes* species, 4490 were identified as *I. ricinus*, 32 as *I. canisuga*, and 4 as *I. hexagonus*. From the identified ticks, 450 pools were selected for molecular analysis out of a total of 2250 identified *I. ricinus* ticks (50.1%). Among these, 431 pools originated from animal hosts and 19 from vegetation. Additionally, analysis was conducted on 6 pools out of 32 collected *I. canisuga* ticks and 1 pool out of 4 identified *I. hexagonus* ticks. 

Pooling criteria were based on geographical origin and representativeness, ensuring an adequate quantity of ticks from various regions of Bosnia and Herzegovina, particularly concerning *I. ricinus* (covering different species of wild and domestic animals, including livestock and pets). Similarly, due to the smaller number of collected *I. canisuga* and *I. hexagonus*, only one pool of *I. hexagonus* and 6 pools of *I. canisuga* were examined. Grouped samples contained up to 5 adult ticks, and sample selection was adjusted based on the number of ticks collected from the same animal or vegetation. In cases where ≥10 adult ticks were collected from a single host or herd, every other tick was selected for sampling. Likewise, if ≥20 ticks were collected under similar conditions, every fourth tick was chosen. Ticks were transferred from individual vials to sterile tubes using fine-tipped forceps, ensuring specimen integrity. The number of ticks per pool was recorded, and tubes were labeled for tracking and analysis. Prepared samples were then stored at −20 °C until further testing.

### 2.3. Molecular Identification of Pathogens

Nucleic acid extraction from samples utilized a QIAamp DNA Mini kit (Qiagen, Hilden, Germany), following the manufacturer’s instructions. Before DNA extraction, all ticks were carefully dissected and halved using sterile scissors, ensuring that sterile conditions were maintained throughout the process. The dissected halves of ticks were individually homogenized in lysis buffer using a TissueLyser II (Qiagen, Hilden, Germany) to effectively disrupt tick tissues and release DNA. Homogenization was conducted at high speed to ensure thorough tissue disruption. Following this, DNA extraction was performed from the homogenized samples using the standard protocol provided by the manufacturer. The remaining dissected tick halves were frozen at deep temperatures as a precaution for potential future studies. DNA amplification was conducted using the QuantiFast Pathogen PCR + IC Kit (Qiagen, Hilden, Germany) on the Mic qPCR Cycler platform (Bio Molecular Systems, Upper Coomera, Queensland, Australia). The specific primers, probes, and relevant information for the investigated pathogens are detailed in Table 1. 

A multiplex PCR approach was employed for selected pathogens, involving two duplex PCRs for *Rickettsia* spp. and *Babesia* spp., as well as *A. phagocytophilum* and *B. burgdorferi* s.l. Identification of *Rickettsia* spp., based on the amplification of a segment of the gltA gene, and *Babesia* spp., based on the amplification of a segment of the 18S rRNA gene, followed established protocols from previous studies [35,36]. Protocols for amplifying the ospA gene segment of *B. burgdorferi* s.l. and Msp2 genes of *A. phagocytophilum* were also based on previously published studies [37,38]. Cycling conditions for both duplex PCRs were identical, involving initial denaturation at 95 °C for 5 min, followed by 40 cycles of denaturation at 95 °C for 15 s, and annealing and elongation at 60 °C for 30 s. Fluorescence reading and graphical plotting were automatically conducted at 60 °C at the conclusion of each extension/elongation phase using micPCR software version 2.8.13 (Bio Molecular Systems, Upper Coomera, Queensland, Australia).

### 2.4. Data Analysis

The prevalence of *Rickettsia* spp., *Babesia* spp., *A. phagocytophilum*, and *B. burgdorferi* s.l. infection in positive adult ticks collected at a certain region was estimated using the Minimum Infection Rate (MIR), i.e., the minimum infected proportion expressed as a percentage: MIR = (p/N) × 100%, where p = the number of positive pools and N = the total number of ticks tested. This method assumes that only one infected tick is present in each positive pool [39,40].

## 3. Results

### 3.1. Tick Species Distribution

**Figure 2:**
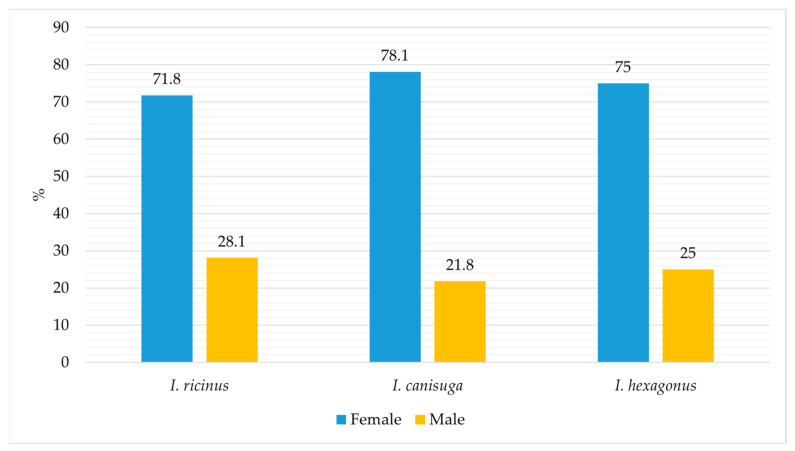
Collected females and males of *I. ricinus*, *I. canisuga*, and *I. hexagonus* ticks.

The proportion of *I. ricinus* out of the total collected adult ticks from 2017 to 2023 was 60.3% (4490/7438). It was 0.4% (32/7438) and 0.05% (4/7438) for *I. canisuga* and *I. hexagonus*, respectively. The percentage of collected females and males of *I. ricinus*, *I. canisuga*, and *I. hexagonus*, relative to the total number of each species collected from all regions, animal species, and vegetation, is shown in Figure 2.

**Figure 3:**
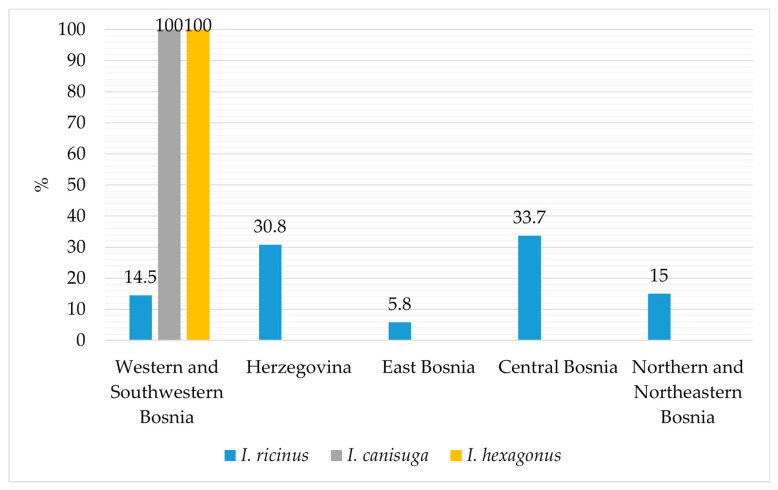
Distribution of collected *I. ricinus*, *I. canisuga*, and *I. hexagonus* according to the hosts and vegetation.

**Figure 4:**
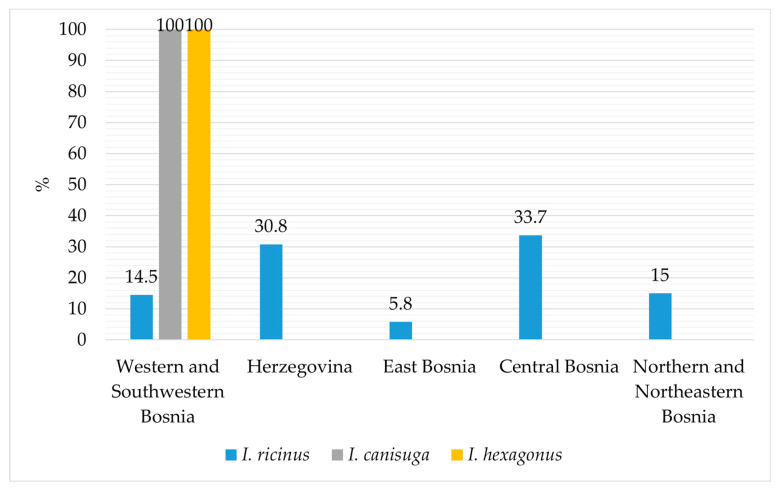
Spatial distribution of *I. ricinus*, *I. canisuga*, and *I. hexagonus* across different regions of Bosnia and Herzegovina.

Figure 3 illustrates the distribution of *I. ricinus*, *I. canisuga*, and *I. hexagonus* according to the hosts and vegetation from which they were collected. *Ixodes ricinus* was predominantly collected from dogs, goats, and cattle, while to a slightly lesser extent from cats, sheep, foxes, roe deer, and vegetation, and from all defined sites in Bosnia and Herzegovina (Figure 3). Similarly, *I. hexagonus* was only collected from dogs, while *I. canisuga* was collected from foxes and dogs, specifically from the areas of Western and Southwestern Bosnia (Figure 4). Most of the collected *Ixodes* ticks were from the Central Bosnia and Herzegovina region.

### 3.2. Pathogen Detection and Identification of Tick Pools

The molecular analysis of 450 *I. ricinus* tick pools revealed an MIR of 22.1%. None of the examined *I. canisuga* and *I. hexagonus* pools tested positive for any of the pathogens analyzed. Detailed information regarding the MIRs (minimum infection rates) of pathogens in *I. ricinus* ticks according to locality and animal host can be found in Table 2. Detailed information regarding the MIRs of pathogens in *I. ricinus* ticks from vegetation according to locality can be found in Table 3.

*Rickettsia* spp. was detected in *I. ricinus* ticks collected from dogs, cats, sheep, and goats from all sites except Eastern Bosnia (Table 2). The overall MIR was 6.3% in pools of all ticks collected from animals across all locations. Specifically, the MIRs for Western and Southwestern Bosnia, Herzegovina, Central Bosnia, Northern and Northeastern Bosnia were 10.4%, 10.6%, 3.6%, and 6.6%, respectively.

*Babesia* spp. was detected in *I. ricinus* ticks from dogs and cattle in Northern and Northeastern Bosnia with an MIR of 3.1% and in ticks collected from vegetation in Western and Southwestern Bosnia with an MIR of 3.6% (Table 3). *Anaplasma phagocytophilum* was confirmed in *I. ricinus* ticks from all locations and hosts, including dogs, goats, and cattle (Table 2). The overall infection rate for *A. phagocytophilum* was 8.8%, the highest among the four pathogens tested. The individual MIRs by locality were 13% in Western and Southwestern Bosnia, 8.2% in Herzegovina, 35.2% in Central Bosnia, 54.4% in Eastern Bosnia, and 7% in Northern and Northeastern Bosnia.

*Borrelia burgdorferi* s.l. was detected in Central Bosnia, Eastern Bosnia, and Northern and Northeastern Bosnia in *I. ricinus* from dogs and cats, with an overall MIR of 3.4% (Table 2). The individual infection rates by locality were 22.5% in Central Bosnia, 24.4% in Eastern Bosnia, and 13.1% in Northern and Northeastern Bosnia. 

A total of 1.1% (5/450) of pooled samples tested positive for at least two investigated pathogens. Co-infection of *B. burgdorferi* s.l. and *A. phagocytophilum* was detected in one pooled sample originating from dogs in the Eastern Bosnia region, while in two pooled samples from dogs in the Herzegovina and Western and Southwestern Bosnia regions, the presence of two pathogens, *Rickettsia* spp. and *A. phagocytophilum*, was confirmed (Table 4). Each tick from these pooled samples was individually retested to confirm the presence of pathogens, revealing three ticks originating from three pooled dog samples as carriers of both pathogens.

Co-infection of *B. burgdorferi* s.l. and *Rickettsia* spp. was detected in one pooled sample originating from cats in Central Bosnia. The ticks from the positive cat pool were individually tested, and within the pool, the presence of both pathogens was confirmed in two ticks. Additionally, in one pooled sample from goats in the Northern and Northeastern Bosnia region, the presence of *Rickettsia* spp. and *A. phagocytophilum* was confirmed (Table 4). Similarly, ticks from the positive pool were individually tested, revealing co-infection in one tick.

## 4. Discussion

Bosnia and Herzegovina exhibit high climatic and habitat heterogeneity conducive to ticks and the spread of TBDs, which is increasingly becoming a concern for veterinary and public health. The results of this study highlight the frequent occurrence of TBPs in *I. ricinus* collected from various domestic animal species and vegetation; given that it is the most prevalent tick species in Bosnia and Herzegovina [22,24], this research holds significant importance. Considering that *I. hexagonus* and *I. canisuga* rarely infest humans and that this study also determined a low frequency of these ticks in both domestic and wild animals, their significance in the context of veterinary and public health is relatively minor. However, they can certainly play an important role as maintenance vectors in silent enzootic transmission cycles. 

This study represents the first comprehensive examination of a larger number of *I. ricinus* ticks collected from domestic and wild animals and vegetation for the pathogens under investigation. Moreover, to the best of the authors’ knowledge, this study represents the first investigation into the presence of pathogens in *I. canisuga* and *I. hexagonus* ticks in Bosnia and Herzegovina. 

Given that the majority of ticks were collected from animals (97.4%) rather than vegetation (2.6%), it is noteworthy that the proportion of females compared to males is higher in all three *Ixodes* species, with 71.8%, 78.1%, and 75% for *I. ricinus*, *I. canisuga*, and *I. hexagonus*, respectively (Figure 2). The overall proportion of *I. ricinus* among all collected tick species was 60.3%, which represents a slightly lower frequency compared to the results of previous studies conducted in Bosnia and Herzegovina, where the overall frequency was reported as 63.8% [22] and 70.5% [24]. Our findings indicate that *I. ricinus* ticks were predominantly identified on dogs, consistent with previous research in Bosnia and Herzegovina [22,24]. However, for the first time, this species was identified on deer and red foxes (Figure 3). *Ixodes ricinus* was most frequently encountered in the regions of Central Bosnia (33.8%) and Herzegovina (31%), followed by the regions of Northern and Northeastern Bosnia (15%) and Western and Southwestern Bosnia (14.5%). In this study, *I. ricinus* was the least prevalent in the Eastern Bosnia region (5.8%) (Figure 4). This distribution pattern is consistent with the results of the earlier investigation conducted in Bosnia and Herzegovina [22,24]. 

Furthermore, this study confirmed the presence of *I. canisuga* and *I. hexagonus* on animals at frequencies of 0.4% and 0.05%, respectively. *Ixodes canisuga* sampled from red foxes and dogs had a frequency of 0.4%, while *I. hexagonus* was found in 0.05% of samples collected from four dogs. Their low frequencies are consistent with previous research on ticks in Bosnia and Herzegovina, where 0.09% of *I. hexagonus* was sampled from dogs, while *I. canisuga* was sampled from red foxes at a frequency of 0.3% [24,25,26]. Both species were sampled in locations within the Western and Southwestern Bosnia region (Figure 4), and their presence was also confirmed in previous studies conducted in the Southwestern Bosnia region [22,24].

DNA from *Rickettsia* spp. was detected in 6.3% (95% CI 5.0–7.5) of *I. ricinus* pools collected from animals. The highest rate of infection was confirmed in the Herzegovina region (10.6%), while no presence of *Rickettsia* spp. was confirmed in ticks from the Eastern Bosnia region. The majority of positive ticks originated from dogs with an MIR ranging from 2.4% in Northern and Northeastern Bosnia to 14.2% in Herzegovina (Table 2). Additionally, the presence of *Rickettsia* spp. was confirmed for the first time in ticks from goats (7.14%) in Northern and Northeastern Bosnia, cats (4.7%) in Central Bosnia, and sheep (9.5%) in Herzegovina (Table 2). In this study, ticks collected from vegetation were not positive for the presence of *Rickettsia* spp. Data about the occurrence of *Rickettsia* in ticks are limited in Bosnia and Herzegovina. However, there are reports of finding *Rickettsia* spp. in ticks collected from vegetation in Bosnia and Herzegovina. Hodžić et al. [28] molecularly identified *R. monacensis* (1.1%) and *R. helvetica* (5.7%) in 21.8% of *I. ricinus* ticks in Bosnia and Herzegovina. Additionally, in studies conducted on people in Bosnia and Herzegovina, antibodies against *Rickettsia* were detected in blood samples from individuals from the northwestern part of Bosnia and Herzegovina. Of the 231 sera tested using the complement fixation test (CFT), 61.5% were positive for *Rickettsia typhi*, 4.3% for *Rickettsia prowazekii*, and 1.7% for *Rickettsia conorii*. Meanwhile, 183 serum samples tested using IFAT showed that 37.7% were positive for *R. typhi* and 1.6% for *R. conorii* [41]. The detection of *Rickettsia* at the recorded infection rates in tick pools (MIRs of 2.4–14.3%), particularly in ticks from dogs, underscores the need for ongoing surveillance and research to monitor and reduce the risks of TBDs in Bosnia and Herzegovina.

In the present study, the MIR for *Babesia* spp. was 3.1% (95% CI 2.2–3.9) in female *I. ricinus* pools. *Babesia* spp. was identified in eight pools (100%; eight/eight) originating from vegetation in Western and Southwestern Bosnia, but it was not detected in tick pools from animals from this region (Table 3). However, the small sample size of ticks from vegetation in this study makes it difficult to draw general conclusions, highlighting the need for a larger investigation. Of the examined ticks from animals in Northern and Northeastern Bosnia, *Babesia* spp. was detected in six *I. ricinus* pools sampled from cattle (17.1%) and forty pools from dogs (16%) (Table 2). Studies on babesiosis in Bosnia and Herzegovina in sheep and cattle were conducted in previous research by examining blood and ticks collected from animals. *Babesia divergens* was confirmed in the blood of clinically ill cattle in central Bosnia using blood smears and molecular detection methods [30,38]. Additionally, in the same study, *B. divergens* was confirmed in two female *I. ricinus* ticks (25%; two/eight) collected from animals [30]. In a study on *Babesia ovis* in sheep blood from 2022 in the area of Eastern Bosnia and Herzegovina, out of 192 tested sheep, *B. ovis* was confirmed in 70 (36.4%). The lack of reports on babesiosis in ruminants in the area of Bosnia and Herzegovina is likely due to the fact that mild and subclinical cases often go undiagnosed [30,38,42]. Given that bovine babesiosis has been present in cattle grazing on pastures where *I. ricinus* is reported as the dominant tick species, it is likely that *Babesia* is maintained in the environment with endemic stability; although, *Babesia* spp. were not detected in ticks originating from wild animals in our study. Previous research confirmed *Babesia vulpes* and *Babesia canis* in spleen samples of red foxes with frequencies of 31.9% and 0.8%, respectively [43]. *Babesia* spp. “Badger type A” was identified in a European wildcat (*Felis silvestris*) in Bosnia and Herzegovina [44]. 

Additionally, as this is the first examination of the presence of *Babesia* spp. from ticks on dogs, it is noteworthy that *Babesia* spp. was not confirmed in *I. ricinus* collected from dogs in Bosnia and Herzegovina. However, in previous research conducted in Bosnia and Herzegovina, *Babesia* spp. has been confirmed in a larger number of studies in blood. Peripheral blood samples taken from one hundred and thirty-four dogs in the northeastern part of Bosnia confirmed the presence of *B. canis* in seven dogs (5.2%) [45]. Furthermore, a study on blood smears from the peripheral blood of 183 dogs in Northern Bosnia confirmed a slightly higher prevalence of *Babesia* spp. infection (30.6%) [46]. A lower prevalence of infection was reported by Hrvat [47], where, in the northeastern part of Bosnia, *Babesia* spp. was identified in 11.8% of 432 dogs. *Babesia canis* was confirmed by molecular detection methods in dog blood in the study by Ćoralić et al. [48], with a frequency of 85% out of 80 dogs with symptoms of babesiosis. Furthermore, *Babesia vogeli* was identified in two dogs in Central Bosnia and one dog in the Western and Southwestern Bosnia region (0.7%) [49].

*Anaplasma phagocytophilum* is recognized as an emerging TBP that is important for animals and humans [50]. This study provides the first confirmation of *A. phagocytophilum* in *I. ricinus* in Bosnia and Herzegovina, with an MIR of 8.8% (95% CI 7.4–10.4). It was detected in all regions of Bosnia and Herzegovina, at various locations and in different animal species. The *A. phagocytophilum*-positive male and female pools were detected in *I. ricinus* sampled from dogs in all regions, except Northern and Northeastern Bosnia, with an infection rate ranging from 0.9% to 23.8% (Table 2). In this study, ticks collected from vegetation were not positive for the presence of *A. phagocytophilum.* In previous studies conducted in Bosnia and Herzegovina, *Anaplasma* was mainly confirmed in the blood of infected animals. Colella et al. [49] collected blood samples from 408 dogs and tested them using a microfluidic RT-PCR test for 43 different pathogens. The study revealed the presence of single and mixed infections. Furthermore, *Anaplasma platys* was confirmed in one dog in the Herzegovina region (0.2%) [49]. In this study, *A. phagocytophilum* was also detected in *I. ricinus* species collected from ruminants for the first time in *I. ricinus* from cattle in Northeastern Bosnia (20%) and goats in Eastern Bosnia (20.4%). Additionally, mixed infection with *Rickettsia* spp. and *A. phagocytophilum* was confirmed in three pools of *I. ricinus* collected from dogs and goats. 

DNA from *B. burgdorferi* s.l. was detected in *I. ricinus* pools collected from dogs and cats in Central Bosnia, Eastern Bosnia, and Northern and Northeastern Bosnia, with an infection rate of 3.4% (95% CI 2.5–4.3). In this study, ticks collected from vegetation were not positive for the presence of *B. burgdorferi* s.l. Additionally, this study confirmed a mixed infection in one tick pool with *B. burgdorferi* s.l. and *A. phagocytophilum* originating from dogs and *B. burgdorferi* s.l. and *Rickettsia* spp. in cats. These findings are in agreement with other studies that confirmed the presence of these pathogens as single and/or coinfection in ticks, dogs, and cats [51]. In previous studies conducted in Bosnia and Herzegovina, Lasić et al. [27] found that out of 48 *I. ricinus* collected from human patients in the Central Bosnia region, 32 (66.7%) tested positive for the *B. burgdorferi* s.l. complex. Over the past 10 years, a slight increase in reported cases of Lyme borreliosis in humans has been observed in Bosnia and Herzegovina. According to statistical data from the Institute of Public Health in Bosnia and Herzegovina, the total number of reported cases of Lyme borreliosis in humans from 2002 to 2018 was 1081 [52,53]. Furthermore, another study on *B. burgdorferi* s.l. and other pathogens in questing ticks was conducted by Hodžić et al. in 2015 [44]. However, none of the 30 examined *I. ricinus* ticks tested positive for *B. burgdorferi* s.l. [44].

Ticks may also carry multiple pathogens simultaneously, increasing the likelihood of co-transmission to their hosts. Given the observed co-infections in individual ticks in our study, our findings emphasize the need for vigilance regarding potential public health or veterinary issues in Bosnia and Herzegovina. Our study involved the individual testing of ticks in cases where co-infections or mutual infections were suspected based on initial sample screening results; however, this approach may have limitations. The selective nature of individual testing could have led to underestimation or missed detection of certain pathogens, particularly those with low prevalence or those not identified during initial sample screening. Therefore, future research should consider the feasibility of comprehensive individual testing of ticks to improve the accuracy and reliability of pathogen detection.

## 5. Conclusions

This is the first comprehensive study of a larger number of *I. ricinus* ticks for various pathogens in the area of Bosnia and Herzegovina, as well as the first investigation of *I. canisuga* and *I. hexagonus*. The results of this study provide valuable insights into the dynamics of TBPs. These findings underscore the importance of broader surveillance and control measures for the spread of *Ixodes* species, both in Bosnia and Herzegovina and in areas with similar ecological conditions. Moreover, the discovery of pathogens in *I. ricinus* raises concerns about their potential transmission to humans as well as the threat to financial losses in livestock production. Given the interconnectedness of ecosystems and the mobility of hosts and vectors, urgent collaboration among researchers, public health authorities, and policymakers is needed to develop and implement integrated strategies for the prevention, surveillance, and control of these diseases.

## Figures and Tables

**Table 1:** Primers and probes used for molecular detection of pathogens (Real-Time PCR).

Pathogen	Primer (100 pmol/µL)	Oligonucleotide Sequence (5’–3’)	Final Conc./25 µL	Amplicon Size (bp)	Ref.
*Rickettsia* spp.	Rspp-F	GAGAGAAAATTATATCCAAATGTTGAT	450 nM	100	34
	Rspp-R	AGGGTCTTCGTGCATTTCTT			
	Rspp-P	[CY5]-CATTGTGCCATCCAGCCTACGGT-[BHQ2]	150 nM		
*Babesia* spp.	Bab-F	CAGCTTGACGGTAGGGTATTGG	1 µM	20	35
	Bab-R	TCGAACCCTAATTCCCCGTTA			
	Bab-P	[YAKYE]- CGAGGCAGCAACGG-[BHQ1]	500 nM		
*B. burgdorferi* s.l.	Bbsl_ospA-F	AATATTTATTGGGAATAGGTCTAA	600 nM	59	36
	Bbsl_ospA-R	CACCAGGCAAATCTACTGA			
	Bbsl_ospA-P	[FAM]-TTAATAGCATGTAAGCAAAATGTTAGCA-[BHQ1]	100 nM		
*A. phagocytophilum*	Apmsp2-F:	ATGGAAGGTAGTGTTGGTTATGGTATT	900 nM	77	37
	Apmsp2-R	TTGGTCTTGAAGCGCTCGTA			
	Apmsp2-P	[CY5]-TGGTGCCAGGGTTGAGCTTGAGATTG-[BHQ2]	125 nM		

**Table 2:** The total number of *I. ricinus* pools tested and MIRs of pathogens detected in tick pools according to the locality and animal host.

	No. of Tested Tick Pools	*Rickettsia* spp.		*Babesia* spp.		*A. phagocytophilum*		*B. burgdorferi* s.l.	
		No. of Positive Pools	MIR (95% CI)	No. of Positive Pools	MIR (95% CI)	No. of Positive Pools	MIR (95% CI)	No. of Positive Pools	MIR (95% CI)
Western and Southwestern Bosnia									
Dogs	90	28 ♀	8.9 (5.8–12.0)	0	0	43 ♀	13.7 (9.9–17.4)	0	0
		13 ♂	4.1 (1.9–6.3)			3 ♂	0.9 (0.0–2.0)		
Cats	4	0	0	0	0	0	0	0	0
Cattle	6	0	0	0	0	0	0	0	0
Sheep	5	0	0	0	0	0	0	0	0
Goats	7	0	0	0	0	5 ♀	20.4 (6.8–40.7)	0	0
Herzegovina									
Dogs	42	21 ♀	14.3 (8.6–19.9)	0	0	17 ♀	11.6 (6.4–16.7)	0	0
Cats	6	0	0	0	0	0	0	0	0
Cattle	6	0	0	0	0	0	0	0	0
Sheep	3	1 ♀	9.5 (0.0–26.1)	0	0	0	0	0	0
Goats	2	0	0	0	0	0	0	0	0
Central Bosnia									
Dogs	59	8 ♀	3.9 (1.2–6.5)	0	0	20 ♀	9.7 (5.6–13.7)	15 ♀	7.3 (3.7–10.8)
						5 ♂	2.4 (0.3–4.5)		
Cats	6	1 ♀	4.8 (0.0–13.9)	0	0	0	0	1 ♀	4.8 (0.0–13.9)
Cattle	2	0	0	0	0	0	0	0	0
Sheep	2	0	0	0	0	0	0	0	0
Goats	2	0	0	0	0	0	0	0	0
Eastern Bosnia									
Dogs	72	0	0	0	0	34 ♀	13.5 (9.3–17.7)	22 ♀	8.7 (5.3–12.2)
Cats	0	0	0	0	0	0	0	0	0
Cattle	18	0	0	0	0	15 ♀	23.8 (14.0–34.3)	0	0
Sheep	0	0	0	0	0	0	0	0	0
Goats	0	0	0	0	0	0	0	0	0
Northern and Northeastern Bosnia									
Dogs	82	15 ♀	5.2 (2.7–7.8)	40 ♀	16 (11.8–20.3)	0	0	13♀	4.5 (2.1–6.9)
		7 ♂	2.4 (0.7–4.2)						
Cats	2	0	0	0	0	0	0	0	0
Cattle	10	0	0	6	17.1 (10.8–21.3)	7 ♀	20 (6.8–33.3)	0	0
Sheep	1	0	0	0	0	0	0	0	0
Goats	4	1 ♀	7.1 (0.0–20.6)	0	0	0	0	0	0
Total	431	95	6.3 (5.0–7.5)	46	3.1 (2.2–3.9)	133	8.8 (7.4–10.4)	51	3.4 (2.5–4.3)

No.—number of tested/positive tick pools; ♀—female ticks; ♂—male ticks; MIR—minimum infection rate (%), CI—Confidence interval.

**Table 3:** The total number of *I. ricinus* pools tested and MIRs of pathogens detected in tick pools from vegetation according to the locality.

	No. of Pools Tested	*Rickettsia* spp.		*Babesia* spp.		*A. phagocytophilum*		*B. burgdorferi* s.l.	
		No. of Positive Pools	MIR (95% CI)	No. of Positive Pools	MIR (95% CI)	No. of Positive Pools	MIR (95% CI)	No. of Positive Pools	MIR (95% CI)
Western and Southwestern Bosnia	8	0	0	8 ♀	100 (100)	0	0	0	0
Herzegovina	5	0	0	0	0	0	0	0	0
Central Bosnia	4	0	0	0	0	0	0	0	0
Eastern Bosnia	0	0	0	0	0	0	0	0	0
Northern and Northeastern Bosnia	2	0	0	0	0	0	0	0	0
Total	19	0	0	8	3.6 (2.3–4.6)	0	0	0	0

No.—number of tested/positive tick pools; ♀—female ticks; MIR—minimum infection rate (%), CI—Confidence interval.

**Table 4:** Co-infection of pooled *I. ricinus* ticks.

Co-Detection of Different Pathogens	No. of Co-Infection Pool, (MIR)	No. of Co-Infection Pool, (MIR)	No. of Co-Infection Pool, (MIR)
	Dogs	Cats	Goat
*B. burgdorferi* s.l. + *A. phagocytophilum*	1 ♀, 0.08	0	0
*B. burgdorferi* s.l. + *Rickettsia* spp.	0	1 ♀, 5.5	0
*Rickettsia* spp. + *A. phagocytophilum*	2 ♀, 0.16	0	1 ♀, 6.6

No.—of co-infection pool; ♀—female ticks; MIR—minimum infection rate (%).

## Data Availability

The data presented in this study are available on request from the corresponding author.
